# From immune equilibrium to tumor ecodynamics

**DOI:** 10.3389/fonc.2024.1335533

**Published:** 2024-05-10

**Authors:** Xiaoping Chen

**Affiliations:** ^1^ State Key Laboratory of Respiratory Disease, Center for Infection and Immunity, Guangzhou Institutes of Biomedicine and Health, Chinese Academy of Sciences, Guangzhou, China; ^2^ CAS Lamvac (Guangzhou) Biomedical Technology Co., Ltd., Guangzhou, China

**Keywords:** immune equilibrium, immunodynamics, tumor ecosystem, tumor ecodynamics, tumor ecological momentum, immunotherapy, immune ecotherapy

## Abstract

**Objectives:**

There is no theory to quantitatively describe the complex tumor ecosystem. At the same time, cancer immunotherapy is considered a revolution in oncology, but the methods used to describe tumors and the criteria used to evaluate efficacy are not keeping pace. The purpose of this study is to establish a new theory for quantitatively describing the tumor ecosystem, innovating the methods of tumor characterization, and establishing new efficacy evaluation criteria for cancer immunotherapy.

**Methods:**

Based on the mathematization of immune equilibrium theory and the establishment of immunodynamics in a previous study, the method of reverse immunodynamics was used, namely, the immune braking force was regarded as the tumor ecological force and the immune force was regarded as the tumor ecological braking force, and the concept of momentum in physics was applied to the tumor ecosystem to establish a series of tumor ecodynamic equations. These equations were used to solve the fundamental and applied problems of the complex tumor ecosystem.

**Results:**

A series of tumor ecodynamic equations were established. The tumor ecological momentum equations and their component factors could be used to distinguish disease progression, pseudoprogression, and hyperprogression in cancer immunotherapy. On this basis, the adjusted tumor momentum equations were established to achieve the equivalence of tumor activity (including immunosuppressive activity and metabolic activity) and tumor volume, which could be used to calculate individual disease remission rate and establish new efficacy evaluation criteria (ieRECIST) for immunotherapy of solid tumor based on tumor ecodynamics. At the same time, the concept of moving cube-to-force square ratio and its expression were proposed to calculate the area under the curve of tumor ecological braking force of blood required to achieve an individual disease remission rate when the adjusted tumor ecological momentum was known.

**Conclusions:**

A new theory termed tumor ecodynamics emphasizing both tumor activity and tumor volume is established to solve a series of basic and applied problems in the complex tumor ecosystem. It can be predicted that the future will be the era of cancer immune ecotherapy that targets the entire tumor ecosystem.

## Highlights

This work advances immunology and oncology in three major ways:

- it has theoretically unified the important concepts and theories of immunology and oncology, such as immune equilibrium, cancer immunoediting, tumor ecosystem, and cancer hallmarks, representing a major progress in the theories of immunology and oncology;
- it advances immunology and oncology from the science of qualitative description to the science of precise quantification; and
- it analyzes the new phenomena caused by the current cancer immunotherapy, solves the major clinical problems of early judgment of pseudoprogression and hyperprogression of tumor, and puts forward a new set of efficacy criteria for cancer immunotherapy, representing a major progress in clinical immunology and clinical oncology.

## Introduction

The theory of immune equilibrium runs through the entire field of immunology and has a history of more than 100 years. It has grasped the interaction between the two essential components of immunity, namely, mutual restriction and balance between positive and negative immunity (1, 2). In the field of tumor immunology, the theory of immune equilibrium also runs through it; however, researchers do not consciously realize that the theory of tumor immunology they have created is essentially derived from the theory of immune equilibrium—for example, the theory of cancer immunoediting proposed by Schreiber et al. in 2002 (3) divides the immune resistance encountered in the development of tumors into three stages: elimination, equilibrium, and escape. The elimination phase is similar to the process of acute pathogen infection, in which the immune system goes through a complete response to eliminate all cancer cells and then returns to a physiological balance state. The equilibrium stage is a chronic physiological immune imbalance state, which can also be understood as a pathological immune equilibrium, that is, a cancer-immune equilibrium (4, 5), and the escape stage can be described as a pathological immune disequilibrium far away from physiological equilibrium or away from pathological equilibrium.

The breadth and depth of the study on the mechanism of tumorigenesis and development seem to go beyond the scope of tumor immunology, thus giving rise to the development of another scientific theory, namely, the theory of tumor ecosystem or tumor ecology (6–8). In addition to the immune system and cancer cells, the tumor ecosystem involves a series of non-immune components. However, if it is assumed that not only cancer cells but also the entire tumor ecosystem hijacks negative immunity to promote its own development, then it is still possible to treat the tumor ecosystem with immunology and the theory of immune equilibrium (which will be discussed systematically in the “Discussion” section). Cancer cells can be considered as new species emerging in the large environment of the host, interacting with cellular or non-cellular components, not only with the local tumor microenvironment (TME) but also with distant tissue and organ niches as well as the nervous, endocrine, and whole immune systems to build a self-sustainable tumor ecosystem (7). Owing to the excessive complexity of this system, in recent years, researchers have adopted advanced techniques such as transcriptomics, especially single-cell transcriptomics, to conduct in-depth studies (9–11). Although the results obtained are exciting, they are quite complex, so it is difficult to use them to guide the clinical practice of cancer treatment. However, if the immune equilibrium theory is applied to the tumor ecosystem, it can be simplified to a stark contrast of two forces: cancer cells and the tumor ecosystem use negative immunity against positive immunity and thus gain the ability to survive and thrive.

Positive immunity and negative immunity are the two most essential components of immune function, and their interaction and mutual restraint are among the most essential core problems in the field of immunology (1, 2, 12, 13). However, the theory describing this core problem, namely, the theory of immune equilibrium, is not the core theory of immunology (1), which forms a great paradox in immunology—namely, the theory of the core problem is not the core theory. The author of this paper recently developed the theory of immune equilibrium into the theory of immunodynamics by mathematizing it, transforming it from a philosophical category into concrete science (14). However, the immunodynamic equations only solve the problem of the measurement of immune response induced by immunotherapy, and they are still unable to solve the problem of the measurement of tumor ecosystems because the tumor ecosystems of different patients require different amounts of immune response in order to benefit patients. The author adopted the method of reverse immunodynamics, that is, the immune braking force is regarded as the tumor ecological force, and the immune force is regarded as the tumor ecological braking force. In other words, the reciprocal form of the immune force equation is regarded as the equation of the tumor ecological force, and the momentum concept of physics is introduced into the tumor ecodynamics—namely, the tumor ecological force (written as *F_ib_*) compared to the speed (transformed from force) of movement of the object, and the tumor volume (denoted as *V*) compared to the quality of the moving object; so, the product of tumor ecological force multiplied by tumor volume is defined as the tumor ecological momentum (denoted as *M_te_*). That is the basic equation for tumor ecological momentum, i.e., $M_{te}=\left(F_{ib}\right)\left(V\right)$ . From this basic equation, a series of new concepts and expressions, namely, a series of tumor ecodynamic equations, are derived, which are then used to solve the dynamic and quantitative problems of the complex tumor ecosystem and to guide the personalized and quantified immunotherapy of cancer.

## Methods

### Reverse immunodynamics

Using the method of reverse immunodynamics, the reciprocal form of the immune force equation (14) is taken as the tumor ecological force equation, namely, the negative immune power (*P_ni_*) as positive tumor ecological power in the equation as the numerator and the positive immune power (*P_pi_*) as the negative tumor ecological power as the denominator in the equation, to obtain the consolidation equation of theoretical and practical tumor ecological force (*F_ib_*) equations, namely, the following Equation (1). Similarly, the method of reverse immunodynamics is used to establish the combined equation of theoretical and practical tumor ecological braking force equations, namely, Equation (2), as follows:


(1) $$F_{ib}=\frac{P_{ni}}{P_{pi}}=\frac{\left(Y1\right)\left(Y2\right)\left(Y3\right)\ldots \left(Yn\right)}{\left(X1\right)\left(X2\right)\left(X3\right)\ldots \left(Xn\right)}=\frac{\left(TGF\beta \right)\left(pSTAT3\right)}{\left(IFN\gamma \right)\left(pSTAT1\right)}$$

(2) $$F_{im}=\frac{P_{pi}}{P_{ni}}=\frac{\left(X1\right)\left(X2\right)\left(X3\right)\ldots \left(Xn\right)}{\left(Y1\right)\left(Y2\right)\left(Y3\right)\ldots \left(Yn\right)}=\frac{\left(IFN\gamma \right)\left(pSTAT1\right)}{\;\left(TGF\beta \right)\left(pSTAT3\right)}$$

It is worth noting that, in immunodynamic equations, there is an adjustment coefficient (*C_oe_*) (14), but in tumor ecodynamic equations, there is no *C_oe_*. This is because, at the beginning, when exploring practical immunodynamic equations, there are many hypothetical equations based on functional data, and in the course of using functional data to select the practical equations that can represent the theoretical equations, the baseline levels of functional data for each hypothetical equation are different, and the introduction of *C_oe_* can make the baseline levels of all assumed equations consistent; they are all equal to 1. Thus, it is convenient to compare different equations and facilitate an intuitive understanding of the immunodynamic equations (14). However, if *C_oe_* is introduced into the tumor ecodynamic equations, it will not only hinder the intuitive understanding of the equations but also increase the computational complexity; more importantly, it would make it impossible to compare different baseline levels in patients with cancers before treatment. Of course, there is no error in logic if we introduce *C_oe_* into these equations, and it is just not easy to understand and use them. In addition, in tumor ecodynamics, there are no negative equations because there is no need to compare positive and negative dynamic curves in the same coordinate system. This is different from the situation in which I first explored immunodynamics.

## Results

### Tumor ecological momentum

If we consider cancer immunotherapy as a breakthrough or revolution in oncology (15), we can say that the traditional methods used to describe cancer and the criteria used to evaluate efficacy have not kept pace with this revolution—for example, Response Evaluation Criteria In Solid Tumors (RECIST) only emphasizes tumor size and ignores tumor activity, and immune-related RECIST (irRECIST) only makes some supplements to RECIST according to the characteristics of immunotherapy (16). In essence, irRECIST is still based on judgment of tumor size, emphasizing its dynamic change process. In contrast, PET Response Criteria In Solid Tumors (PET-based PERCIST) (17) emphasizes only tumor activity and ignores tumor size. According to the new concept of tumor ecodynamics, I propose that, to meet the requirements of the era of cancer immunotherapy, we must emphasize both tumor activity and tumor size and define the product of tumor activity multiplied by tumor volume as tumor ecological momentum. The methodology of calculating tumor ecological momentum can solve new problems that cannot be solved by traditional methods—for example, the delayed effects of immunotherapy, as opposed to the immediate response to conventional chemotherapy, targeted drug therapy, radiation therapy, and surgery, and the persistence of its therapeutic effect after the end of treatment confused the traditional efficacy evaluation criteria (including RECIST and irRECIST). Although pseudoprogression related to immunotherapy (18) can be confirmed by follow-up (irRECIST criteria), it cannot be determined at the end of treatment. Moreover, traditional methods are ineffective for early identification and prediction of hyperprogression (19). The essence of this phenomenon is that “tumor volume” is a “dead” concept, which only contains the resting value and not the active or dynamic value of the tumor. The tumor *F_ib_* value can instead represent tumor immunosuppressive activity, which is a measure of underlying tumor dynamics or motion. Here we borrow the concept from physics: the mass of a moving object multiplied by its velocity equals the momentum of the object. If the tumor size (tumor volume, *V*) compared to the mass of the object, the tumor activity such as tumor *F_ib_* value (force) compared to the speed of movement of the object, then the product of both can be defined as the tumor ecological momentum (denoted as *M_te_*). The general expression formula of *M_te_*, namely the basic equation for tumor ecodynamics, is the following Equation (3). There may be *n* tumor lesions in one tumor-bearing host; the *F_ib_* values of these lesions can be denoted as *F_ib_*_1_, *F_ib_*_2_, *F_ib_*_3_… *F_ibn_*, respectively; the volumes of the lesions can be denoted as *V*_1_, *V*_2_, *V*_3_…*V_n_*, respectively; and the total *M_te_* (*M_tet_*) value of these lesions is the sum of each *F_ib_* multiplying its related *V*, which can be expressed by the following Equation (4). It needs to be stressed that Equation (4) in the present case is only applicable to an animal tumor model study; it does not apply to clinic because in clinic it is almost impossible to get all the tumor lesions to detect their *F_ib_* values. In this case, we can choose one of the most dominant lesions as the representative, and this lesion, called main lesion, can be the largest or primary lesion in the body, which should be determined by clinicians according to its importance and feasibility of clinical manipulations. The tumor ecological momentum, *F_ib_* value and tumor volume of the main lesion are denoted as *M_tem_*, *F_ibm_*, and *V_m_*, respectively. Therefore, the expression of *M_tem_* can be represented by the following Equation (5). At the same time, active tumor could release *F_ib_* into the blood stream. If *F_ib_* was an inorganic small molecule, it would be diluted by the blood of the whole body; thus, using the current detection technology might not be able to test it from the bloodstream. However, *F_ib_* is not an inorganic small molecule; it is instead a set of molecules with complex bioactivity—for example, cancer cells or tumor tissues release TGFβ into the blood through exosomes (cancer-derived exosomes) (20), thereby activating the pSTAT3 signal in blood cells, and TGFβ and pSTAT3 are signaling molecules upstream and downstream of each other (21, 22), so they will induce a positive feedback response and last for a certain period of time. With the progression, especially to the advanced stage of the tumor, not only the TGFβ released from the tumor tissue into the blood will be greatly increased but also the tumor cells themselves will enter the blood and become circulating tumor cells (CTCs), resulting in tumor metastasis (23). TGFβ derived from tumor tissue and CTCs activate platelets in the blood, which (platelets) also express TGFβ and adhere to the surface of CTCs and wrap the CTCs to protect them from attack by immune cells (24, 25). In addition, activated platelets will secrete a large amount of TGFβ which further inhibits immune cells in the blood and even inhibits the function of the entire immune system, thus inhibiting the antitumor immune response of the body (25)—that is to say, the blood *F_ib_* (denoted as *F_ibb_*) contains the information of activity of solid tumors. In this way, we can use the patient’s *F_ibb_* multiplied by the patient’s total blood volume (written as *V_b_*) to obtain the tumor ecological momentum of blood (written as *M_teb_*). It is notable that *V_b_* is a constant, not a variable, that is, it will not change following immunotherapy. Knowing *V_b_* to be a constant is enough; it is unnecessary to compute the concrete numerical value because it will disappear automatically in the equation that is going to calculate the individual disease remission rate in the later section. *F_ib_* can be broken down into two factor pairs, namely, the factor pair TGFβ/INFγ and factor pair pSTAT3/pSTAT1. Two factors in the same factor pair must be tested using the same method and expressed by the same unit to ensure the correctness of Equations (1) and (2) (14). *F_ib_* is a pure ratio, with no unit, but it represents a kind of “dynamic” (immunosuppressive activity) value. In clinic, tumor volume (*V*) can be unified using cubic centimeter. The unit of *M_te_* is momentum cube centimeter (mcc) or moving cube (mc) for short. We can use the following way to describe *M_tem_* and *M_teb_*: “Through calculation, the patient’s *M_tem_* is 92 (mc), and *M_teb_* is 1.5 *V_b_* (mc)”.


(3) $$M_{te}=\left(F_{ib}\right)\left(V\right)=\left(\frac{\left(TGF\beta \right)\left(pSTAT3\right)}{\left(IFN\gamma \right)\left(pSTAT1\right)}\right)\left(V\right)$$

(4) $$M_{tet}=\sum^{\;}\{\left(F_{ib1}\right)\left(V_{1}\right)+\left(F_{ib2\;}\right)\left(V_{2}\right)+\;(F_{ib3})\left(V_{3}\right)\ldots (F_{ibn})\left(V_{n}\right)\}$$

(5) $$M_{tem}=\left(F_{ibm}\right)\left(V_{m}\right)=\left(\frac{\left(TGF\beta_{m}\right)\left(pSTAT3_{m}\right)}{\left(IFN\gamma_{m}\right)\left(pSTAT1_{m}\right)}\right)\left(V_{m}\right)$$

(6) $$M_{teb}=\left(F_{ibb}\right)\left(V_{b}\right)=\left(\frac{\left(TGF\beta_{b}\right)\left(pSTAT3_{b}\right)}{\left(IFN\gamma_{b}\right)\left(pSTAT1_{b}\right)}\right)\left(V_{b}\right)$$

A similar equation based on PET imaging can also be applied clinically. Cancer cells prioritize the use of glucose for glycolysis under aerobic conditions, and their ability to use glucose far exceeds that of normal cells. This phenomenon is known as the Warburg effect (26). The Warburg effect can also be defined as the metabolic activity of a tumor. Both the metabolic activity (27–29) and immunosuppressive activity of tumors contain TGFβ and pSTAT3 signals; therefore, the tumor standardized uptake value (SUV) of glucose detected by PET imaging technology has a similar function to the tumor *F_ib_* value. However, the current PET technology system has no concept of tumor ecological momentum. In this paper, I suggest that the product of the SUV value of the tumor multiplied by its volume represents the approximate value of the tumor ecological momentum. Clinically, PET is usually used for whole-body examination, and it is relatively easy to obtain the volume and SUV values of all measurable lesions in the body; therefore, recalculating the tumor ecological momentum of the main lesion is unnecessary. To distinguish the *F_ib_*-based *M_te_* and *M_tet_* from the PET-based tumor ecological momenta, the latter are denoted as *M_TE_* and *M_TET_*. Based on the analysis above, the general formula for the calculation of PET-based tumor ecological momentum, namely, Equation (7), and the specific calculation formula, namely, Equation (8), as shown below, can be obtained. As for the SUV in these two equations, whether to use the average or maximum SUV for tumors is left to PET specialists to study.


(7) $$M_{TE}=\left(SUV\right)\left(V\right)$$

(8) $$M_{TET}=\sum^{\;}\left\{\left(SUV_{1}\right)\left(V_{1}\right)+\left(SUV_{2}\right)\left(V_{2}\right)+\left(SUV_{3}\right)\left(V_{3}\right)\ldots \left(SUV_{n}\right)\left(V_{n}\right)\right\}$$

In Equations (5), (6), and (8), the tumor activity indices *F_ibm_*, *F_ibb_*, and *SUV*, as well as the tumor ecological momentum indicators *M_tem_*, *M_teb_*, and *M_TET_*, and the blood *F_imb_* [from the *F_im_* of Equation (2)] after the start of immunotherapy can be used to judge or predict disease progression, pseudoprogression, and hyperprogression (19)—for example, if a patient’s tumor volume (*V*) increases during or after immunotherapy, progression, pseudoprogression, or hyperprogression can be determined using these indicators. The criteria used are listed in **Table 1**.

**Table 1:** Key parameters of tumor ecodynamics for judging and predicting disease progression, pseudoprogression, and hyperprogression.

V↑	*F_imb_*	*F_ibm_*	*F_ibb_*	*SUV*	*M_tem_*	*M_teb_*	*M_TET_*
Progression	↓/=	↑	↑	↑	↑	↑	↑
Pseudoprogression	↑	↓	↓	↓	↓/=	↓/=	↓/=
Hyperprogression	↓	↑↑	↑↑	↑↑	↑↑	↑↑	↑↑

↓/=, decrease or no change; ↑, increase; ↑↑, substantial increase; ↓, decrease.

### Adjusted tumor ecological momentum

The equations of tumor ecological momentum above have a flaw, which can be found in the following example: If a cancer patient receives immunotherapy treatment and the activity of all the tumor lesions in the body is eliminated, imaging (such as CT) shows that the tumor volumes are basically unchanged. This indicates that the tumors probably died, leaving scar tissues at the tumor sites. Theoretically, the tumor ecological momentum in this case should be equal to zero. However, when Equations (5), (6), and (8) are used for the calculation, the tumor ecological momentum is not equal to zero. This is because the *F_ib_* and *SUV* values of the scar tissues or normal tissues in the original locations of the tumors are not equal to zero. This non-zero value is called the background value. The difference between the actual detected *F_ib_* value or *SUV* value minus the background value is defined as the adjusted *F_ib_* value or adjusted *SUV* value. The adjusted *F_ib_* is denoted as ${F^{\prime }}_{ib}$ , and the adjusted *SUV* is denoted as *SUV’*. Similarly, the adjusted *M_te_* is denoted as ${M^{\prime }}_{te}$ , and the adjusted *M_TE_* is denoted as ${M^{\prime }}_{TE}$ . The background value of *F_ib_* is denoted as $F_{ib}^{b}$ , and the background value of *SUV* is denoted as *SUV^b^*. Thus, we have the equations: ${F^{\prime }}_{ib}={F^{\;}}_{ib}-F_{ib}^{b}$ and $SUV^{\prime }=SUV-SUV^{b}$ as well as ${M^{\prime }}_{te}=({F^{\prime }}_{ib})(V)$ and ${M^{\prime }}_{TE}=(SUV^{\prime })(V)$ . Therefore, when the detected value and background value are equal, namely, when ${F^{\prime }}_{ib}=0$ or $SUV^{\prime }=0$ , no matter how much the volume (*V*) of the tumor is, its adjusted tumor ecological momentum ( ${M^{\prime }}_{te}$ ) is equal to zero. The reverse is also true, regardless of the activity ( ${F^{\prime }}_{ib}$ or $SUV^{\prime }$ ) of the tumor; as long as *V *= 0, the adjusted tumor ecological momentum ( ${M^{\prime }}_{te}$ ) must be equal to zero. Through this process, the activity (*F_ib_* or *SUV*) of the tumor is equivalent to the volume (*V*) of the tumor. This provides a mathematical basis for the calculation of the individual disease remission rate described in the section below. According to the tumor ecological momentum Equations (3), (8) and (5), (6) above, the corresponding adjusted tumor ecological momentum calculation formulae can be obtained, namely, Equations (9)–(12).


(9) $$M_{te}^{'}=\left(F_{ib}^{'}\right)\left(V\right)=\left(F_{ib}-F_{ib}^{b}\right)\left(V\right)$$

(10) $$\begin{array}{c} M_{TET}^{'}=\sum \left\{\left(SUV_{1}^{'})(V_{1})+(SUV_{2}^{'}(V_{2})+(SUV_{3}^{'})(V_{3})\ldots (SUV_{n}^{'}(V_{n}\right)\right\}=\\ \sum \{(SUV_{1}-SUV_{1}^{b})(V_{1})+(SUV_{2}-SUV_{2}^{b})(V_{2})+(SUV_{3}-SUV_{3}^{b})(V_{3})\ldots \\ (SUV_{n}-SUV_{n}^{b})(V_{n})\} \end{array}$$

(11) $$M_{tem}^{'}=\left(F_{ibm}^{'}\right)\left(V_{m}\right)=\left(F_{ibm}-F_{ibm}^{b}\right)\left(V_{m}\right)$$

(12) $$M_{teb}^{'}=\left(F_{ibb}^{'}\right)\left(V_{b}\right)=\left(F_{ibb}-F_{ibb}^{b}\right)\left(V_{b}\right)$$

Currently, we need to investigate the peripheral blood, various organs, and tissues of normal laboratory animals (on the premise of no immune response) to determine their $F_{ib}^{b}$ values. We can calculate the ratio between the $F_{ibb}^{b}$ values of the peripheral blood and those of various organs and tissues in normal laboratory animals, such as blood–brain ratio, blood–lung ratio, and blood–liver ratio. At the same time, we need to determine the $F_{ib}^{b}$ value of peripheral blood in healthy people, which is an easy thing to do. However, testing for $F_{ib}^{b}$ values of various organs and tissues in healthy persons may be difficult or may not be allowed according to medical ethics. If this is the case, data from laboratory animals should be used—for example, using the known ratios between blood and various organs/tissues in laboratory animals and the known value of peripheral blood in healthy people, we can estimate the $F_{ib}^{b}$ values of various organs and tissues in healthy people. The author suggests a physical examination project to carry out blood $F_{ibb}$ detection. On one hand, $F_{ibb}$ may be a cancer marker and may help identify early cancer; on the other hand, once the person being tested develops cancer much later, the current test data can also be used as his or her $F_{ibb}^{b}$ value. Physicians can use this value and the blood–organ/tissue ratios to calculate the $F_{ib}^{b}$ values of various organs and tissues of the patient. In the PET technology system, there is a solution regarding the background value *SUV^b^*, which should not be described here.

The adjusted tumor ecological momentum equations mentioned above can be used to calculate the individual disease remission rate described below, thus helping to establish new efficacy evaluation criteria adapted to immunotherapy, as well as to determine the progression, pseudoprogression, and hyperprogression of tumors. However, the (unadjusted) tumor ecological momentum equations are only suitable for judging progression, pseudoprogression, and hyperprogression and are not suitable for calculating the individual disease remission rate.

### Individual disease remission rate

In traditional efficacy evaluation criteria, such as RECIST (30), irRECIST (16), and PERCIST (17) (see footnote in **Table 2**), qualitative or semi-quantitative terms, such as complete response (CR), partial response (PR), stable disease (SD), and progressive disease (PD), are used to estimate efficacy. However, in terms of tumor ecodynamics, although the methods above can still be referred to in the stage of immature conditions, I suggest using a fully quantitative individual disease remission rate (remission rate) after the condition is mature as a new set of therapeutic efficacy evaluation criteria. The remission rate (*R_te_*) is defined as the ratio of the difference of $M_{te}^{'}$ before treatment (written as $M_{te}^{'\Delta }$ ) minus $M_{te}^{'}$ after treatment (written as $M_{te}^{'\nabla }$ ) to $M_{te}^{'\Delta }$ ; the general formula is Equation (13) (shown below). According to the same principle, the remission rates based on different adjusted tumor ecological momenta ( $M_{TET}^{'}$ , $M_{tem}^{'}$ , and $M_{teb}^{'}$ ) are obtained respectively, namely *R_TET_*, *R_tem_*, and *R_teb_*, and their calculation formulae are as follows: Equations (14)–(16). For example, in a cancer patient, before immunotherapy, the $M_{tem}^{'\Delta }$ value is 100 (mc), and after completion of the treatment, the $M_{tem}^{'\nabla }$ value is 50 (mc), and the remission rate *R_tem_* = (100–50)/100 = 0.5 (50%). However, if $M_{tem}^{'\nabla }=0$ after treatment, *R_tem_* = (100 − 0)/100 = 1 (100%). If $M_{tem}^{'\nabla }=150$ after treatment, *R_tem_* = (100–150)/100 = – 0.5 (–50%). We can also calculate the comprehensive remission rate (*R_com_*) using *R_TET_*, *R_tem_*, and *R_teb_*. In essence, *R_com_* is the mean value of all or two of *R_TET_*, *R_tem_*, and *R_teb_* [see Equation (17) below].

**Table 2:** Outlines of ieRECIST.

*CR_TET_*	*PR_TET_*	*PD_TET_*
*R_TET_* = 1	0 ≤ *R_TET_* < 1	*R_TET_* < 0
*CR_tem_*	*PR_tem_*	*PD_tem_*
*R_tem_* = 1	0 ≤ *R_tem_* < 1	*R_tem_* < 0
*CR_teb_*	*PR_teb_*	*PD_teb_*
*R_teb_* = 1	0 ≤ *R_teb_* < 1	*R_teb_* < 0
*CR_com_*	*PR_com_*	*PD_com_*
*R_com_* = 1^*^	0 ≤ *R_com_* < 1	*R_com_* < 0

CR = Complete Remission; *PR* = Partial Remission; *PD* = Progressive Disease. Particularly needed to note that *PMR*, *PMR*, and *PMD* are used correspondingly in PERCIST, but not used in ieRECIST.

* If only one of the remission rates, *R_TET_, R_tem_*, and *R_teb_* equals 1, the condition can be judged as "Probable Complete Remission" (*PCR*), such as *PCR_TET_, PCR_tem_*, and *PCR_teb_*, which can be applied to *PR* and *PD*, such as *PPR_TET_, PPR_tem_, PPR_teb_, PPD_TET_, PPD_tem_*, and *PPD_teb_*.


(13) $$R_{te}=\frac{M_{te}^{'\Delta }\;-\;M_{te}^{'\nabla }}{M_{te}^{'\Delta }}$$

(14) $$R_{TET}=\frac{M_{TET}^{'\Delta }\;-\;M_{TET}^{'\nabla }}{M_{TET}^{'\Delta }}$$

(15) $$R_{tem}=\frac{M_{tem}^{'\Delta }\;-\;M_{tem}^{'\nabla }}{M_{tem}^{'\Delta }}$$

(16) $$R_{teb}=\frac{M_{teb}^{'\Delta }\;-\;M_{teb}^{'\nabla }}{M_{teb}^{'\Delta }}=\frac{\left(F_{ibb}^{'\Delta }\right)\left(V_{b}\right)\;-\;\left(F_{ibb}^{'\nabla }\right)\left(V_{b}\right)}{\left(F_{ibb}^{'\Delta }\right)\left(V_{b}\right)}=\frac{F_{ibb}^{'\Delta }\;-\;F_{ibb}^{'\nabla }}{F_{ibb}^{'\Delta }}=1-\frac{F_{ibb}^{'\nabla }}{F_{ibb}^{'\Delta }}$$

(17) $$R_{com}=\frac{R_{TET}\;+\;R_{tem\;}+\;R_{teb}}{3}\approx \frac{R_{TET}\;+\;R_{tem\;}}{2}\approx \frac{R_{TET}\;+\;R_{teb\;}}{2}\approx \frac{R_{tem}\;+\;R_{teb\;}\;}{2}$$

It is worth noting that Equation (15) can be used to prove $M_{tem}^{'}$ and $M_{tet}^{'}$ being roughly equivalent. $M_{tem}^{'}$ is a part of $M_{tet}^{'}$ , so the latter must be *n* times (*n* ≥ 1) of the former, namely, $M_{tet}^{'\Delta }=(n)(M_{tem}^{'\Delta })$ . In principle, if immunotherapy is administered systemically (intravenously, intramuscularly, etc.), the effects of the therapy are roughly evenly distributed to the main lesion and the cluster of other lesions. Therefore, in general, the trend and degree of changes induced by treatment in the main lesion and the cluster of other lesions (but not each lesion) are roughly equivalent. Mathematically, this “roughly equivalent” is expressed using the approximately equal sign “≈,” namely, $M_{tet}^{'\nabla }\approx (n)(M_{tem}^{'\nabla })$ . Based on Equation (15), it is obtained that $R_{tet}=(M_{tet}^{'\Delta }\;-M_{tet}^{'\nabla })/M_{tet}^{'\Delta }\approx \left\{\left(n)(M_{tem}^{'\Delta })-(n)M_{tem}^{'\nabla }\right)\right\}/\left\{(n)(M_{tem}^{'\Delta })\;\right\}\approx (M_{tem}^{'\Delta }-M_{tem}^{'\nabla })/M_{tem}^{'\Delta }\approx R_{tem}$ . Therefore, in terms of the remission rate, $R_{tet}\approx R_{tem}$ and $M_{tem}^{'}\approx M_{tet}^{'}$ . The results show that, in the clinic, if patients receive immunotherapy treatment using systemic methods, it is not necessary to compute *M_tet_*, $M_{tet}^{'}$ , and *R_tet_*. If the calculation is necessary, we have to calculate the parameters *M_TET_*, $M_{TET}^{'}$ , and *R_TET_* using PET technology. It is important to note that in the process of the logical operation of Equation (16), the constant *V_b_* disappears automatically. Therefore, when calculating the remission rate, $F_{ibb}^{'}$ and $M_{teb}^{'}$ are completely equivalent, and the equivalence is not affected by treatment methods—that is, it is suitable for all cancer therapies and not only for solid tumors but also for blood cancers.

Based on the remission rates described above, the Response Evaluation Criteria in Solid Tumors for immunotherapy based on tumor ecodynamics (ieRECIST) can be established when the conditions are mature (see **Table 2**). It should be noted that there is no SD in ieRECIST because if there is no treatment or if treatment is ineffective, the disease should be progressive (because the tumor is “in motion”); so, no progress is effective, and this situation belongs to PR. Within PR, there are a series of concrete remission rates, such as *R_com_* remission rate of 50%. Therefore, the ieRECIST is a combination of qualitative and quantitative efficacy evaluations. At present, this set of criteria is mainly applicable to solid tumors. The principles above can also be used for hematologic tumors (especially leukemia and myeloma without tumor volume)—for example, Equations (6), (12), and (16) can be used to develop new therapeutic efficacy evaluation criteria combined with traditional parameters, such as blood and bone marrow images. This work is left to hematologic oncologists.

### Moving cube-to-force square ratio

Based on the research above, the author further proposes a new concept, namely, the concept of a moving cube-to-force square ratio, which is defined as the ratio between the adjusted tumor ecological momentum prior to immunotherapy ( $M_{te}^{'\Delta }$ ) and the area under the curve of the tumor ecological braking force of blood (*AUC F_imb_*) induced by immunotherapy when a given remission rate is achieved. Because the unit of $M_{te}^{'\Delta }$ is the moving cube centimeter (mcc and mc) and the unit of *AUC F_imb_* is the force square centimeter (fsc and fs), it is called the moving cube-to-force square ratio or the cube to square ratio (*R_cs_*). The general formula for *R_cs_*, namely, Equation (18), and the specific formulae, namely, Equations (19)–(21), are shown below.


(18) $$R_{cs}\;\left(R_{te}\right)\;=M_{te}^{'\Delta }/AUC\;F_{imb}\to AUC\;F_{imb}\;\left(R_{te}\right)\;=M_{te}^{'\Delta }/R_{cs}$$

(19) $$R_{cs}\;\left(R_{TET}\right)\;=M_{TET}^{'\Delta }/AUC\;F_{imb}\to AUC\;F_{imb}\;\left(R_{TET}\right)\;=M_{TET}^{'\Delta }/R_{cs}$$

(20) $$R_{cs}\;\left(R_{tem}\right)=M_{tem}^{'\Delta }/AUC\;F_{imb}\to AUC\;F_{imb\;}\left(R_{tem}\right)\;=M_{tem}^{'\Delta }/R_{cs}$$

(21) $$\;R_{cs}\;\left(R_{teb}\right)=M_{teb}^{'\Delta }/AUC\;F_{imb}\to AUC\;F_{imb}\;\left(R_{teb}\right)=M_{teb}^{'\Delta }/R_{cs}$$

The equations above are not traditional functional equations because there is no $R_{te}$ (or a specific $R_{te}$ ) on the right side of the equal sign of the equations. Therefore, the $R_{te}$ in the equations is not a variable but an artificially set remission rate, such as 0.5 (50%) or 1 (100%). The arrows above represent equivalent transformations of the equations. It should be noted that $R_{cs}$ must be used in conjunction with a specific $R_{te}$ '—for example, $R_{cs}$ in $R_{cs}\;\left(R_{tem}\right)$ must be used with $R_{tem}$ . To make it easier to understand, this is an example. A cancer patient had a $M_{tem}^{'\Delta }$ value of 500 (mc) before the treatment of immunotherapy and a $M_{tem}^{'\nabla }$ value of 250 (mc) after treatment; thus, the treatment reduced the $M_{tem}^{'}$ value by 50%, namely, $R_{tem}=0.5$ , and it was calculated at the end of treatment that $AUC\;F_{imb}=200$ (fs). By introducing these values into Equation (20), it was determined that $R_{cs}\;\left(R_{tem}\right)=R_{cs}\;\left(R_{tem}\;0.5\right)=500/200=2.5$ . Then, what does $R_{cs\;}\left(R_{tem}\;0.5\right)=2.5$ mean? This means that if a patient has a pretreatment $M_{tem}^{'\Delta }$ of 500 (mc), the patient needs an $AUC\;F_{imb\;}$ of 500/2.5=200 (fs) to obtain a 50% $R_{tem}$ remission rate. Therefore, the significance of these $R_{cs}$ equations lies in the fact that a specific $M_{te}^{'\Delta }$ value is known, and if a certain remission rate is achieved, the desired $AUC\;F_{imb\;}$ value should be calculated using the equivalent equation on the right of the arrow. However, for a specific case, his or her $R_{cs\;}$ value is not known before treatment, which requires a lot of clinical research and clinical practice to obtain an empirical range of $R_{cs\;}$ value for similar cases and then, based on this range, to estimate how much $AUC\;F_{imb}$ value a new similar patient needs to achieve a given remission rate after treatment (such as $R_{tem}=0.5$ ) — for example, if the value range of $R_{cs}\;\left(R_{tem}\right)=R_{cs\;}\left(0.5\right)=2\sim 3$ for stage IV lung adenocarcinoma is determined through clinical studies and practice, that is, before immunotherapy, patients with stage IV lung adenocarcinoma already have an empirical value range $\;\;R_{cs}\;\left(R_{tem}\right)=R_{cs}\;\left(0.5\right)=2\sim 3$ ; coupled with an exact $M_{tem}^{'\Delta }$ value before treatment, it is possible to estimate the $\mathrm{AUC}\;F_{imb}$ value required for treatment to reach $R_{tem}=0.5$ . If a patient has stage IV lung adenocarcinoma, the empirical value range of $R_{cs}\;\left(R_{tem}\right)=R_{cs}\left(0.5\right)=2\sim 3$ can be used, and the patient needs the value range of $AUC\;F_{imb}=500/2\sim 3=167\sim 250$ (fs) to achieve a remission rate of $R_{tem}=0.5.$ It should be emphasized that, to simplify the mathematics, it is necessary to specify that when making the curve of $F_{imb}$ , the length of 1 cm in the horizontal direction of the coordinate represents the time of 1 day (24 h). Otherwise, more complex calculations must be performed to solve the conversion between the different units. Only in this manner can $R_{cs}$ become a simple ratio without a unit. According to the equation above, the greater the $M_{te}^{'\Delta }$ value before immunotherapy, the greater the $AUC\;F_{imb}$ value required to achieve the treatment effect for a given remission rate. Therefore, if the tumor ecological momentum is reduced by surgery or precision radiotherapy (or any treatment method that does not significantly impair immune function) before immunotherapy, the $AUC\;F_{imb}$ value required to achieve a certain therapeutic effect will be significantly reduced, and the $AUC\;F_{imb}$ value itself depends on the dose and course of immunotherapy. In other words, the smaller the $M_{te}^{'\Delta }$ value before immunotherapy, the lower the dose or shorter the course of treatment required to achieve a certain therapeutic efficacy. By referring to the calculation method of $R_{com}$ , the comprehensive (mean) $AUC\;F_{imb}$ value can be calculated, namely, the calculation formula of $AUC\;F_{imb}\;\left(R_{com}\right)$ is Equation (22) as shown below.


(22) $$\begin{array}{c} AUC\;F_{imb}(R_{com})\\ =\frac{AUC\;F_{imb}(R_{TET})+AUC\;F_{imb}(R_{tem})+AUC\;F_{imb}(R_{teb})}{3}\\ \approx \frac{AUC\;F_{imb}(R_{TET})+AUCF_{imb}(R_{tem})}{2}\\ \approx \frac{AUC\;F_{imb}(R_{TET})+AUC\;F_{imb}(R_{teb})}{2}\\ \approx \frac{AUC\;F_{imb}(R_{tem})+AUC\;F_{imb}(R_{teb})}{2} \end{array}$$

What needs to be pointed out is that if we use the reciprocal of the cube-to-square ratio, namely, the square-to-cube ratio, then the latter is more intuitive than the former, and it is also possible to approximately use the unadjusted tumor ecological momentum $M_{te}$ , so the ratio becomes $\;AUC\;F_{imb}/M_{te}^{\Delta }$ , specifically $AUC\;F_{imb}/M_{tem}^{\Delta }$ , or even $AUC\;F_{imb}/F_{ibb}^{\Delta }$ . These ratios can be used to roughly predict or evaluate the efficacy of immunotherapy: the higher the $\;AUC\;F_{imb}/M_{te}^{\Delta }$ value, the better the efficacy. It is suggested that these indicators can be used in the early stage of the study of tumor ecodynamics.

**Figure 1:**
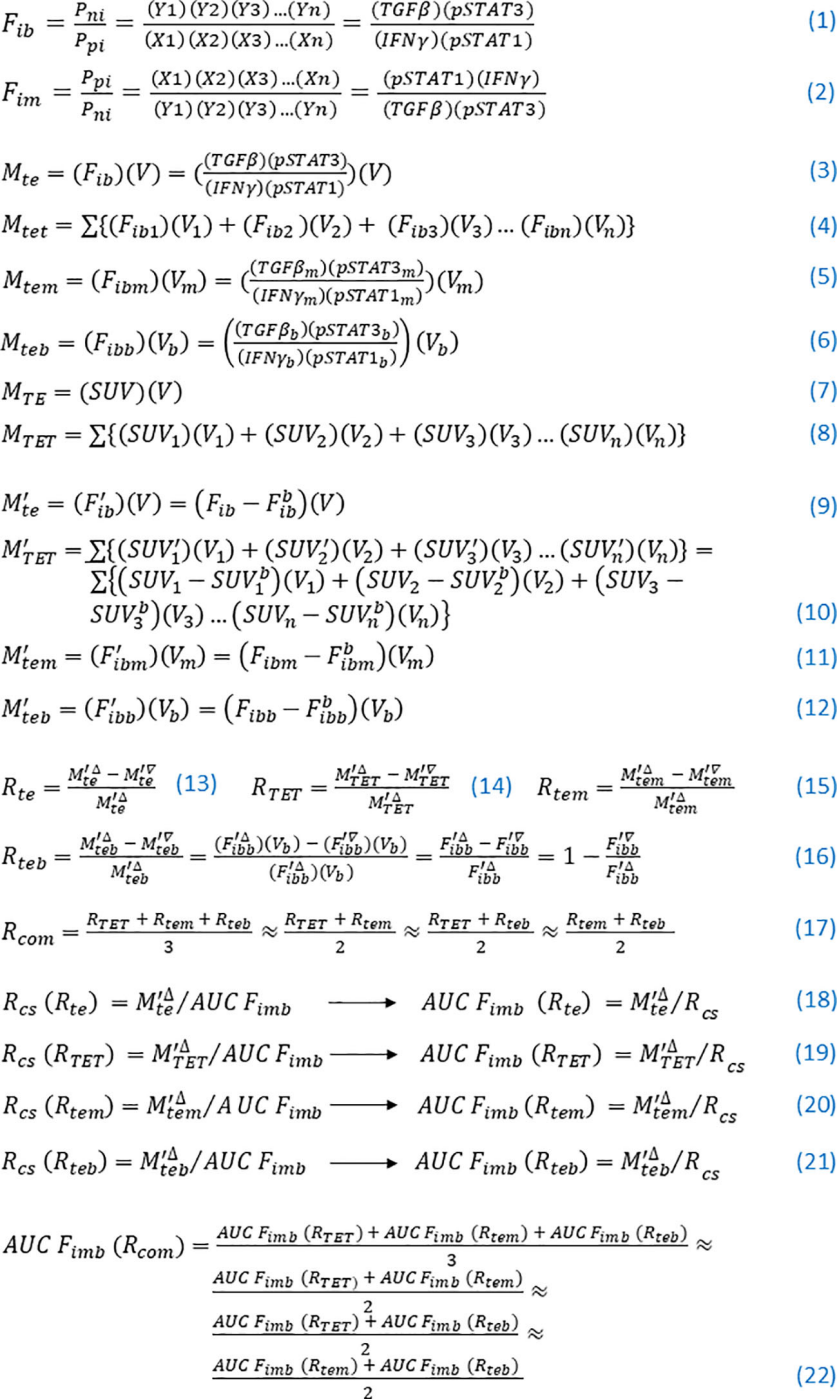
Key concepts and equations of tumor ecodynamics. (1) Tumor ecological force and its expression formula. (2) Tumor ecological braking force and its expression formula. (3)–(8) Tumor ecological momenta and their expression formulae. (9)–(12) The tumor ecological momenta and their expression formulae were adjusted. (13)–(17) Remission rates and their expression formulae. (18)–(21) Moving cube-to-force square ratios and their expression formulae achieve a given remission rate, as well as their transformed equations. (22) The comprehensive (mean) value of the area under the curve of the tumor ecological braking force of blood required to achieve a certain therapeutic effect and its calculation formula.

In summary, a series of concepts and equations of tumor ecodynamics are established based on reverse immunodynamic equations for solving the theoretical and practical problems of the complex tumor ecosystem, which are summarized in **Figure 1** for easy understanding, memorization, and application.

## Discussion

In a previous study, the theory of immune equilibrium was mathematized, and a series of immunodynamic equations were established to solve the quantitative problem of immune response induced by immunotherapy (14). However, the quantitative problem of the complex tumor ecosystem cannot be solved, so only half of the problem of cancer immunotherapy measurement is solved, while the other half needs to be solved by the tumor ecodynamic equations. In this study, the reverse immunodynamics method was adopted to convert the immune braking force into the tumor ecological force and the immune force into the tumor ecological braking force, thus obtaining two reverse immunodynamic equations, namely, Equations (1) and (2). Then, by borrowing the concept of momentum from physics and introducing tumor volume *V* into Equation (1), the basic equation of tumor ecological momentum, namely, Equation (3), is obtained. The prototype for Equation (3) is a very simple formula, namely, $M_{te}=\left(F_{ib}\right)\left(V\right)$ . This theoretically gives tumor an important motion meaning: they can grow if left untreated. By this way, we obtain a series of equations for the tumor ecological momentum, namely, Equations (3)–(6). Since pSTAT3 and TGFβ induce metabolic reprogramming of cancer cells and stromal cells of tumor (27–29), PET is used clinically to examine the metabolic activity of tumor. However, only the metabolic activity of tumor, i.e., SUV value, is used clinically. This is a flaw both in theory and in practice. Equations (7) and (8) proposed in this paper can make up for this defect. Equations (3)–(8) above and the tumor activity indicators ( $F_{ib}$ and SUV ) can be used to determine the progression, pseudoprogression, and hyperprogression of tumor after immunotherapy (**Table 1**) and can be immediately applied in the clinic. The main mechanism of immunotherapy is to carry out indirect effects on cancer cells through activating effector immune cells and/or inhibiting immunosuppressor cells rather than the immediate direct killing of cancer cells (31); therefore, the inhibition of activity and effect of indirect killing often coexist—for example, the entry of activated immune cells into the tumor tissue, resulting in inflammation and edema within the tumor, would lead to an increase in tumor size (18), but at this time the immunosuppressive activity of the tumor tissue should have decreased (due to increased pSTAT1 and IFNγ and/or decreased TGFβ and pSTAT3). As a result, the tumor ecological momentum would remain unchanged or decrease. In addition, IFNγ secreted by activated immune cells can stimulate the expression of pSTAT1 in cancer cells (32). Moreover, the battle between immune cells and cancer cells or tumor stromal cells is protracted (33), which is different from the immediate and direct killing effect of traditional cancer therapies. Therefore, the efficacy evaluation of immunotherapy needs to consider both tumor activity and tumor volume. If immunotherapy induces a $F_{ibb}$ rising or $F_{imb}$ reducing reaction process, it can be immediately judged as hyperprogression of disease, and treatment should be terminated immediately, which has an important clinical guiding significance for the early identification of hyperprogression. However, the equations above related to tumor ecological momentum cannot solve the calculation problem of individual disease remission rate, so the author further proposes the concepts of adjusted tumor ecological momenta and their expressions, namely, Equations (9)–(12). These adjusted tumor ecological momenta can be used to calculate the remission rates after immunotherapy, namely, Equations (13)–(17). Based on the concepts of remission rates and their expressions, the author proposes to implement a new set of efficacy evaluation criteria (ieRECIST) for immunotherapy of solid tumor based on tumor ecodynamics when the conditions are mature (**Table 2**)—that is, in the future, the term individual disease remission rate could be used to describe treatment outcomes. Through the mechanistic analysis of immunotherapy and mathematical logic deduction, the following conclusion is drawn: In terms of calculating remission rates, $M_{tem}^{'}$ and $M_{tet}^{'}$ are roughly equivalent for immunotherapy given systemically, which provides a scientific basis for the reasonableness of simplifying clinical procedures (only the main lesion needs to be considered). An important inference based on the adjusted tumor ecological momentum is that the tumor activity (such as $F_{ib}$ ) is equivalent to the tumor volume. To date, no other studies have shown that other tumor indicators are equivalent to tumor volume. With the popularization and application of cancer immunotherapy, we can predict that there will be more and more long-term survivors with tumor, but there is at least a part of these survivors may have been cured because their tumor activity may have disappeared, namely, $M_{te}^{'}=0$ , only leaving a scar at the site of the tumor. Moreover, even if $M_{te}^{'}\neq 0$ , but just below a certain threshold, the immune system can control the tumor so that the tumor does not develop, thus achieving long-term survival with tumor. In order to further develop the theoretical and practical value of tumor ecodynamics, the author puts forward the important concepts of moving cube-to-force square ratios and their expressions, namely, Equations (18)–(22). At the same time, through mathematical operation, it is proved that $F_{ibb}^{'}$ and $M_{teb}^{'}$ are completely equivalent in calculating the remission rate $R_{teb}$ , which provides a scientific basis for simplifying the computation process of tumor ecodynamics. With the development of tumor ecodynamics, it will be possible in the future to use the formula of moving cube-to-force square ratio to calculate how many doses or how long a course of treatment is required, namely, how much $AUC\;F_{imb}$ value of immunotherapy to achieve a given therapeutic effect (such as a 50% remission rate). By then, cancer immunotherapy will enter a new era of highly individualized and quantifiable immunotherapy. It can be predicted that because the application of tumor ecodynamic indicators of blood [ $F_{imb}$ , $F_{ibb}$ , $F_{ibb}^{'}$ , $M_{teb}$ , $M_{teb}^{'}$ , $R_{teb}$ , $R_{cs}(R_{teb}),AUCF_{imb}(R_{teb})$ , and so on] does not require tumor specimens and PET equipment, they can be rapidly popularized in large-scale clinical research and practice.

**Figure 2:**
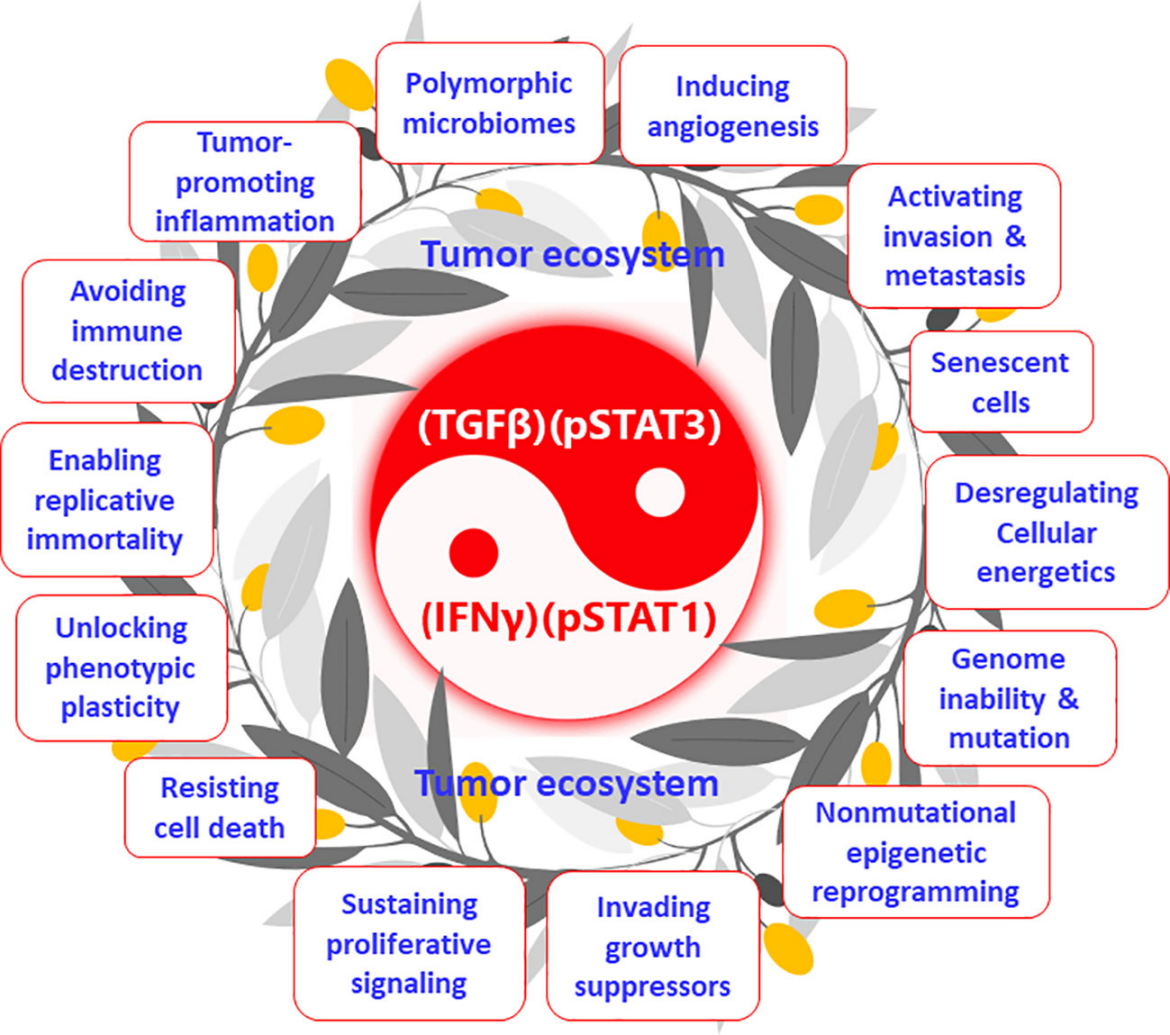
Tumor ecosystem and its functional phenotypes (cancer hallmarks): the tumor ecodynamic model. The tumor ecosystem drives its formation and development by (TGFβ)(pSTAT3) overcoming (IFNγ)(pSTAT1), exhibiting the 14 hallmarks of cancer. This mechanism is in line with the balance theory of Yin (negative regulation) and Yang (positive regulation) in traditional Chinese medicine.

The tumor ecosystem and the tumor macroenvironment (34) proposed in recent years are essentially two expressions of the same concept, both of which break through the concept of tumor microenvironment (TME, only within the tumor) and emphasize the wholeness and participation of all systems in the body. Few researchers have noticed an essential link between the tumor ecosystem and cancer hallmarks. Somarelli proposed that the hallmarks of cancer can be thought of as ecologically driven phenotypes (35). More directly, I propose that cancer hallmarks are phenotypes of the tumor ecosystem (**Figure 2**). Hanahan and Weinberg initially proposed six hallmarks of cancer (36), which were recently developed to 14, including “sustaining proliferative signals” and “invading growth suppressors” (37) (**Figure 2**). Wang et al. systemically described that the occurrence and development of the above-mentioned 14 cancer hallmarks are derived from the activation of STAT3 signaling (38). Furthermore, Mortezaee and Majidpoor stated that TGFβ is a cardinal factor for the induction of all tumor/cancer hallmarks (39). Indeed a series of studies have clarified that TGFβ plays the same or similar roles as STAT3 in the occurrence, development, and metastasis of tumors (40). At the same time, STAT3 and TGFβ interact very closely and are upstream and downstream signals of each other (21, 22). Therefore, they can form a positive feedback loop and generate a vicious cycle. STAT3 and its active form, pSTAT3, are intracellular signaling molecules that act only within cells, whereas TGFβ can transmit signals within and between cells (22); accordingly, TGFβ plays an important role in coordinating the joint actions of the tumor ecosystem—for example, bispecific antibodies that target both TGFβ and PD-L1 can overcome TGFβ-mediated immunotherapy resistance (41). Similarly, pSTAT1 is an intracellular signaling molecule, whereas IFNγ can transmit signals within and between cells; therefore, IFNγ can transmit activation signals within and outside the immune system (32). Although numerous studies have demonstrated that IFNγ is the upstream signaling molecule of pSTAT1 (32, 42), my group’s previous work indicated that pSTAT1 expression peaked on the first day of infection with malarial parasites (43), whereas other groups demonstrated that IFNγ did not peak until the fifth day of *Plasmodium* infection (44). These results suggest that IFNγ and pSTAT1 may also be upstream and downstream signaling molecules of each other in the body, which is worthy of a further study. In a previous study of immunodynamics, I explained that (TGFβ)(pSTAT3), through overcoming (IFNγ)(pSTAT1), promoted the occurrence and development of multiple cancer characteristic events (the term “hallmarks” was not used at the time) (14). Therefore, in immunodynamics, (TGFβ)(pSTAT3) represents negative immune power, and (IFNγ)(pSTAT1) represents positive immune power; conversely, in tumor ecodynamics, (TGFβ)(pSTAT3) represents positive tumor ecological power, and (IFNγ)(pSTAT1) represents negative tumor ecological power. Mechanistically, the tumor ecosystem drives its formation and development by (TGFβ)(pSTAT3) overcoming (IFNγ)(pSTAT1), exhibiting the 14 hallmarks of cancer. The tumor ecosystem is essentially a concept of structure and composition, and cancer hallmarks are the functional phenotypes of this system. Consequently, the ratio ( $F_{ib}$ ) of these two powers can be understood as the original force of occurrence and development of the tumor ecosystem. This mechanism is in line with the balance theory of Yin (negative regulation) and Yang (positive regulation) in traditional Chinese medicine (45). The operating mechanism of tumor ecodynamics is summarized in **Figure 2**. It should be emphasized that the immunodynamic or tumor ecodynamic four-factor relationship (IFNγ)(pSTAT1)/(TGFβ)(pSTAT3) or (TGFβ)(pSTAT3)/(IFNγ)(pSTAT1) is a collective linkage behavior, not a single factor action or one-to-one interaction. If viewed from a single dynamic factor, each factor may have positive and negative functions—for instance, IFNγ has both antitumor and protumor activities (42), and its exact function needs to be determined according to different preconditions (such as different times). This case can be found in my previous study of immunodynamics, namely, *Plasmodium* infection activates IFNγ and pSTAT1 signaling and then induces TGFβ and pSTAT3 signaling through immune balancing mechanisms, with only a time difference between the former and the latter. This time difference is reflected in the $F_{im}$ curve of immunodynamics/tumor ecodynamics, where $F_{im}$ value rises rapidly early in the immune response, falls slowly when it peaks, and eventually returns to baseline level (14).

**Figure 3:**
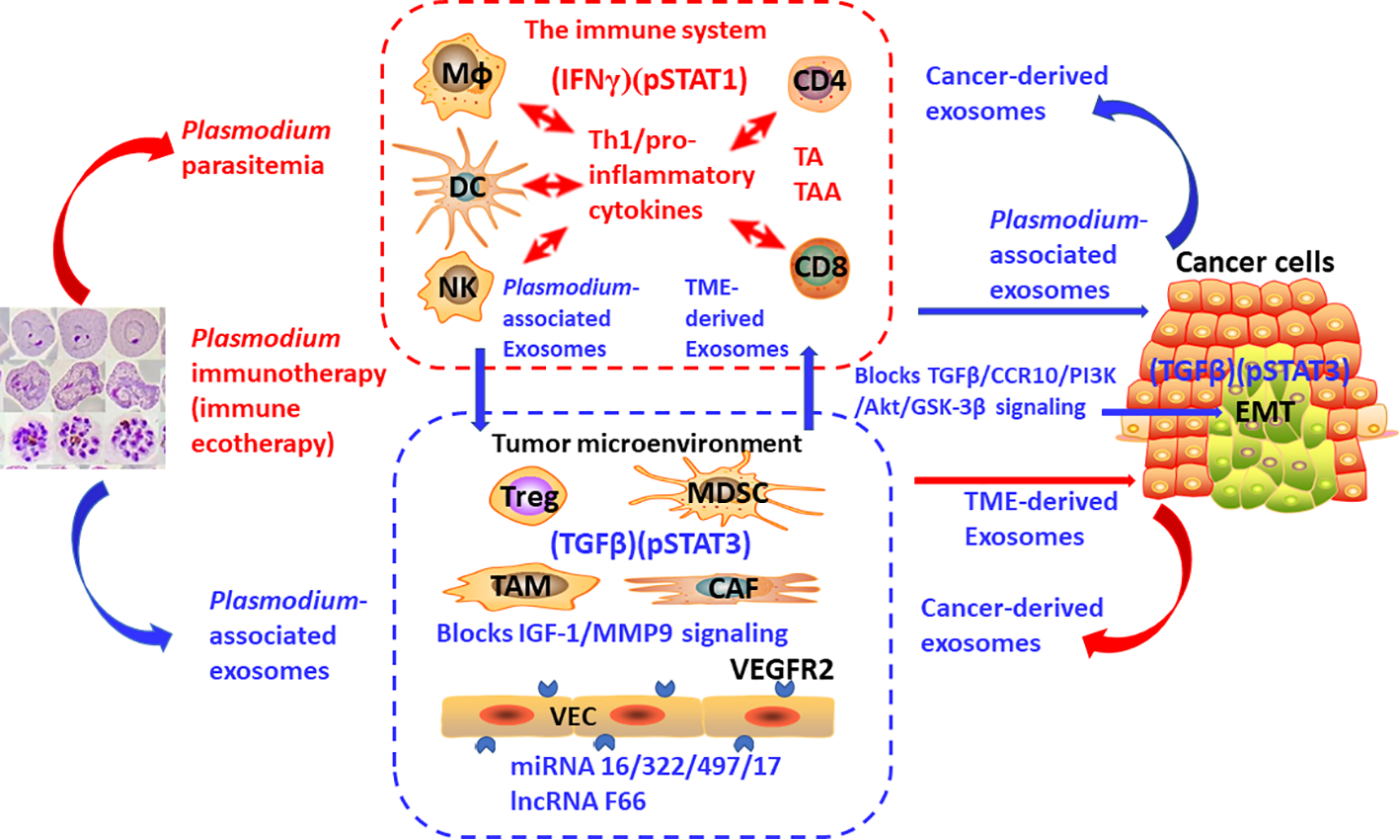
The mechanisms of action of *Plasmodium* immunotherapy (immune ecotherapy) based on tumor ecodynamics. The suppressed immune system by tumor, immunosuppressive tumor microenvironment, and cancer cells constitute one of the basic units that make up the tumor ecosystem, with many identical or similar basic units constituting the entire tumor ecosystem. *Plasmodium* immunotherapy (immune ecotherapy) activates the immune system through IFNγ and pSTAT1 signaling and targets the entire tumor ecosystem through inhibiting TGFβ and pSTAT3 signals. The red arrow represents activation, and the blue arrow represents suppression.

Importantly, at least one cancer immunotherapy by activating (IFNγ)(pSTAT1) and inhibiting (TGFβ)(pSTAT3) is currently in preclinical and clinical studies (NCT02786589, NCT03474822, NCT03375983, and NCT05924776). My team has been working on the research of *Plasmodium* infection against cancer (46). Our previous studies have shown that *Plasmodium* infection (persistent parasitemia) activates the immune system of tumor-bearing hosts (47, 48). The first step is to activate innate immune cells, including NK cells and dendritic cells (DCs), which release Th1-type/pro-inflammatory cytokines, including IFNγ, which, in turn, further activate innate immune cells. Activated innate immune cells kill some of the cancer cells. Dying cancer cells release tumor antigen (TA) or tumor-associated antigen (TAA). In the presence of Th1 cytokines during the infection, these tumor-specific antigens activate CD4+ and CD8+ T cells, which infiltrate the tumor and kill cancer cells more effectively (47). Our studies have demonstrated that a benign form of *Plasmodium* infection activates the immune system through strong pSTAT1 (43) and IFNγ (47) signaling. We also found that *Plasmodium* infection inhibits tumor cells from secreting a series of cytokines and chemokines that recruit immunosuppressor cells, such as myeloid-derived suppressor cells (MDSC), regulatory T cells (Treg), tumor-associated macrophages (TAM), and cancer-associated fibroblast (CAF), through *Plasmodium*-associated exosomes. Thus, the number of immunosuppressor cells in the TME is significantly reduced, and their function is inhibited, resulting in downregulated expression levels of TGFβ and IL-10 in tumor tissues, and therefore the immunosuppressive TME is systematically relieved (46, 49, 50). In the presence of Th1 cytokines and the disarming of the immunosuppressive TME, the activated effector immune cells infiltrate the tumor tissue without a hitch, making the cold tumor hot or hot tumor even hotter (46). Therefore, compared with immune checkpoint inhibitors such as PD-1 antibody, *Plasmodium* immunotherapy may be more likely to induce inflammation and edema within the tumor, resulting in a greater likelihood of pseudoprogression, so it should be more necessary to use ieRECIST based on tumor ecodynamics for efficacy evaluation. In *Plasmodium*-infected mice, the expression level of PD-1 on effector CD8+ T cells infiltrated into tumor tissues is significantly downregulated, while the expression levels of perforin and granzyme B in CD8+ T cells are significantly upregulated. These phenomena may be associated with the inhibition of TGFβ and pSTAT3 signaling in the tumor tissues (49) (and Tao, PhD thesis, 2023). In addition, at least four microRNAs, namely, miRNAs 16/322/497/17 are found within *Plasmodium*-associated exosomes, and a new long non-coding RNA, called lncRNA F63, has been identified in *Plasmodium*-infected tumor tissues. All these RNAs target the VEGFR2 gene in tumor vascular endothelial cells. As a result, this gene is unable to express, so the formation of tumor blood vessels is inhibited (51, 52). *Plasmodium* infection also inhibits tumor angiogenesis by blocking the IGF-1/MMP9 signaling pathway of TAMs in tumor tissue through the metabolite hemozoin of the parasite (50). *Plasmodium* infection blocks the TGFβ/CCR10/PI3K/Akt/GSK-3β signaling pathway, thereby inhibiting epithelial–mesenchymal transition (EMT) of the cancer cells and preventing tumor metastasis and recurrence (53, 54). Moreover, the material basis for suppressing EMT also exists within *Plasmodium*-associated exosomes (unpublished data). Other studies have shown that cancer cells secrete cancer-derived exosomes and that immunosuppressor cells in the TME secrete TME-derived exosomes to suppress immune function, promote tumor angiogenesis, and promote EMT (20, 55, 56). Moreover, our further study has shown that *Plasmodium* immunotherapy combined with conventional radiotherapy cures 70% of glioma (a typical cold tumor) in the brains of mice with no recurrence after 210 days (equivalent to 21 years of observation in clinical trials) (57). Taken together, our series of studies have demonstrated that *Plasmodium* infection activates the IFNγ (47) and pSTAT1 (43) signals of the immune system while suppressing the TGFβ and pSTAT3 signals in tumor tissue (49) (and Tao, PhD thesis, 2023), such that the infection stimulates a tit-for-tat and life-or-death war between the immune system and the tumor ecosystem. The battle between the two forces at the heart of it is, in immunodynamic terms, a campaign between positive immune power (IFNγ)(pSTAT1) and negative immune power (TGFβ)(pSTAT3), or in tumor ecodynamic terms, it is a contest between negative tumor ecological power (IFNγ)(pSTAT1) and positive tumor ecological power (TGFβ)(pSTAT3). **Figure 3** outlines the tumor ecodynamics-based mechanisms of *Plasmodium* immunotherapy. Accordingly, *Plasmodium* immunotherapy can also be called immune ecotherapy because it targets the entire tumor ecosystem through activating the immune system. This is consistent with our previous view that cancer is an ecological disease and that *Plasmodium* immunotherapy is an ecological counterattack therapy (46). It can be predicted that the future cancer treatment should move from single-target immunotherapy to systemic immune ecotherapy. In addition, the emergence and development of immune ecotherapy have the potential to overcome various drug resistance problems to achieve a “sustainable treatment plan” for advanced cancer—for example, one of the resistance mechanisms of traditional therapies is to induce the EMT of cancer cells (58), while one of the resistance mechanisms of current immunotherapies (such as the treatment with PD-1 antibody) is to induce more immunosuppressor cells to enter tumor tissues, thus strengthening the immunosuppressive TME (46). Because immune ecotherapy works by inhibiting EMT and relieving the immunosuppressive TME, if current therapies are interchangeably used with (future) immune ecotherapy (sequential treatment), drug resistance can be overcome and a sustainable treatment plan can be achieved.

Future immune ecotherapy requires more accurate efficacy evaluation using the tumor ecodynamic approach (ieRECIST)—for example, my team, in collaboration with clinical teams, has applied immune ecotherapy (*Plasmodium* immunotherapy) in clinical studies to treat more than 100 patients with advanced solid tumors, showing initial safety and efficacy (46). Among them, there was a case of advanced lung cancer patient who underwent immune ecotherapy, and the morphology of the primary lung lesion changed. The CT scan showed that the lung tumor was “crab-like” (with unclear boundaries and pseudopodia) before treatment and became “patchy” (with clear boundaries and no pseudopodia) after treatment, but the tumor volume did not change significantly. After consultation with the thoracic surgeon, surgical indication was found after treatment (while there was no surgical indication before treatment), and minimally invasive resection of the lesion was recommended. The patient accepted the doctor’s suggestion to undergo minimally invasive surgery. It was found that the tumor was enveloped by a capsule and there was no vascular distribution on the surface. The pathological sections showed that there was a large number of immune cells infiltrating inside the tumor tissue, including a large number of CD3 staining positive T cells. According to the theory of tumor ecodynamics, a large number of (effector) immune cells infiltrating tumor tissue will lead to a decrease in tumor *F_ib_* value. According to the equation $M_{te}=\left(F_{ib}\right)\left(V\right)$ , although the tumor volume (*V*) remained unchanged, the tumor ecological momentum $M_{te}$ would also decrease. Therefore, tumor ecodynamics-based ieRECIST can more accurately reflect the efficacy of immune ecotherapy or immunotherapy than the traditional RECIST or irRECIST. However, it is a pity that the patient in this case did not have tumor samples before treatment, so a comparison of tumor ecodynamic indexes before and after treatment could not be performed. By the way, this patient has been tumor-free for more than 6 years (unpublished data).

Although there are currently some methods for immune score (59) or metabolic-tumor-stroma score (60) of tumor tissues, these methods are only semi-quantitative and not fully quantitative, whereas tumor ecodynamic methods are fully quantitative. It can be predicted that, with research progress in tumor ecodynamics, the three stages of cancer immunoediting can be accurately described quantitatively in the future. It is worth mentioning that a mathematical model has been used to study the dynamics of a tumor–immune ecosystem, but this model is limited to predicting the likelihood of radiotherapy-induced pan-cancer cure (61). There is also a series of mathematical model studies describing the evolutionary dynamics of cancer cells and stromal cells (62, 63) as well as the recent emergence of mathematical model studies for cancer immunotherapy (64, 65). However, these studies lacked a quantitative description of the occurrence and development of the entire tumor ecosystem. These studies all used sophisticated mathematical methods that are difficult for clinicians to understand and apply. The tumor ecodynamics established here should be the first comprehensive theory to quantitatively describe the occurrence and development of the tumor ecosystem, and the basic principle of this theory is simple and clear. It can be said that just a knowledge of high school mathematics and appropriate knowledge of immunology and oncology are required to understand and apply tumor ecodynamics established in this paper.

## Conclusion

It is reasonable to assume that the entire tumor ecosystem hijacks negative immunity to promote its own development; therefore, it is scientific to treat the tumor ecosystem with the theory of immune equilibrium. Based on a previous study of immunodynamics established by mathematizing the theory of immune equilibrium and by using the method of reverse immunodynamics, a series of concepts and equations of tumor ecodynamics are established. These equations are used to address a range of important theoretical and practical problems related to cancer immunotherapy, such as early identification of progression, pseudoprogression, and hyperprogression of tumors, establishment of new efficacy evaluation criteria for immunotherapy of solid tumors based on tumor ecodynamics (ieRECIST), and guidance of individualized immunotherapy. At the same time, the theory of cancer immunoediting is incorporated into the framework of the immune equilibrium theory, which is pushed to the stage of fully quantified tumor ecodynamics. Furthermore, the concepts of tumor ecosystem and cancer hallmarks are also unified into the category of tumor ecodynamics. Therefore, the tumor ecodynamics established here represents an unprecedented new theory in oncology and immunology. However, this work is only the beginning of the study of tumor ecodynamics, and a series of studies is needed to promote its development in the future.

## Data availability statement

The original contributions presented in the study are included in the article/supplementary material. Further inquiries can be directed to the corresponding author.

## Author contributions

XC: Conceptualization, Formal analysis, Investigation, Methodology, Project administration, Validation, Writing – original draft, Writing – review & editing.
